# HPV E6/E7 mRNA Testing Is More Specific than Cytology in Post-Colposcopy Follow-Up of Women with Negative Cervical Biopsy

**DOI:** 10.1371/journal.pone.0026022

**Published:** 2011-10-06

**Authors:** Sveinung Wergeland Sørbye, Marc Arbyn, Silje Fismen, Tore Jarl Gutteberg, Elin Synnøve Mortensen

**Affiliations:** 1 Department of Clinical Pathology, The University Hospital of North Norway, Tromsø, Norway; 2 Unit of Cancer Epidemiology, Scientific Institute of Public Health, Brussels, Belgium; 3 Department of Microbiology and Infection Control, The University Hospital of North Norway, Tromsø, Norway; 4 Department of Medical Biology, Faculty of Health Sciences, University of Tromsø, Tromsø, Norway; Health Canada, Canada

## Abstract

**Background:**

In Norway, women with negative or low-grade cervical biopsies (normal/CIN1) are followed up after six months in order to decide on further follow-up or recall for screening at three-year intervals. A high specificity and positive predictive value (PPV) of the triage test is important to avoid unnecessary diagnostic and therapeutic procedures whereas a low risk of high-grade disease among triage negative women assures safety.

**Materials and Methods:**

At the University Hospital of North Norway, cytology and the HPV mRNA test PreTect HPV-Proofer, detecting E6/E7 mRNA from HPV types 16, 18, 31, 33 and 45, are used in post-colposcopy follow-up of women with negative or low-grade biopsy. In this study, women with negative biopsy after high grade cytology (ASC-H/HSIL) and/or positive HPV mRNA test in the period 2005–2009 were included (n = 520). Histologically confirmed cervical intraepithelial neoplasia of grade 2 or worse (CIN2+) was used as study endpoint.

**Results:**

Of 520 women with negative or low-grade biopsy, 124 women (23.8%) had CIN2+ in follow-up biopsy. The sensitivity and specificity of the HPV mRNA test were 89.1% (95% CI, 80.1–98.1) and 92.5% (95% CI, 88.2–96.7), respectively. The ratios of sensitivity, specificity and PPV of HPV mRNA testing compared to repeat cytology for finding CIN2+ was 1.05 (95% CI: 0.92–1.21), 1.21 (95% CI: 1.12–1.32), and 1.49 (95% CI: 1.20–1.86), respectively. The PPV of mRNA was 77.3% (95% CI, 59.8–94.8) in women aged 40 or older.

**Conclusion:**

Women with negative cervical biopsy require follow-up before resumption of routine screening. Post-colposcopy HPV mRNA testing was as sensitive but more specific than post-colposcopy cytology. In addition, the HPV mRNA test showed higher PPV. A positive mRNA test post-colposcopy could justify treatment in women above 40 years.

## Introduction

Cervical cancer is the third most common cancer affecting women worldwide [1]. In Norway, about 300 women get cervical cancer annually and 80–100 die from the disease [2]. Infection with Human Papillomavirus (HPV) is a necessary cause of cervical cancer [3]. In Norway, a cervical cancer screening program was introduced in 1995, recommending all women between 25 and 69 years of age to have a cytological cell sample (Pap-smear) every three years [4]. The rationale of cervical cancer cytological screening is to identify and treat high-grade cervical intraepithelial neoplasia (CIN) (precancerous lesions) in order to prevent its progression to invasive cancer. Since the introduction of the program, the coverage of women taking a Pap-smear has increased and consequently, the rate of cervical cancer is reduced [5]. Women with cytological diagnoses of either high grade squamous intraepithelial lesion (HSIL) or atypical squamous cells, cannot rule out a high grade lesion (ASC-H) are referred to colposcopy and biopsy [4], as also recommended in European guidelines [6], [7]. The same is the case in women with atypical squamous cells of undetermined significance (ASC-US) or low-grade squamous intraepithelial lesion (LSIL) and positive HPV test [8].

Unfortunately, colposcopy does not have optimal sensitivity for CIN2+. The National Health Service Cervical Screening Programme (NHSCSP) Guidelines for Colposcopy and Programme Management, which guides British practice, ask for evidence of a colposcopic accuracy of 65% [9]. Zuchna et al reported 66.2% sensitivity of CIN2+ when up to three guided cervical biopsies were taken regarded as a diagnostic test with the cone specimen as reference standard [10]. Using digitized cervical images from 919 women referred for equivocal or minor cytologic abnormalities into the ASCUS-LSIL Triage Study, Massad et al reported 39% sensitivity for CIN2+ [11]. Hence, all women with negative colposcopy and biopsies after abnormal cytology and/or HPV-testing have to be followed. In Norway cytology and HPV testing are used in post-colposcopy follow-up of women with negative biopsy (normal or CIN1 histology) [8].

Several HPV tests for use in triage and follow-up are available and different hospitals in Norway use different HPV tests. One of these tests is the PreTect HPV-Proofer assay, detecting HPV E6/E7 mRNA from the five most prevalent subtypes causing cervical cancer. The University Hospital of North Norway (UNN) has five years of experience with the use of this test [8], [12]. The argument for choosing this test in triage of minor cervical lesions and follow-up after negative biopsy was the need for a clinically specific test to find the women truly needing referral to colposcopy, biopsy and treatment. The HPV E6/E7 mRNA test has been shown to have a higher clinical specificity and positive predictive value (PPV) than HPV DNA tests [13]–[18] and thus qualifies better than HPV DNA tests for this purpose. In post-colposcopy follow-up of women with negative biopsy a triage test with high specificity is important to reduce the number of unnecessary rebiopsies and avoid the adverse effects of overdiagnosis and consequent overtreatment [19], [20].

In three previous papers [8], [12], [21] we have reported targeted prediagnostic management of women with minor cytological abnormalities at screening. Now our hypothesis is that HPV E6/E7 mRNA testing is more specific than follow-up cytology without loss in sensitivity in postdiagnostic management of women who have a negative biopsy.

## Materials and Methods

The Regional Committee for Research Ethics (REK Sør-Øst C) has approved the study. Written consent from the patients for their information to be stored in the hospital database and used for research was not needed because the data were obtained and analyzed anonymously. The ethics committee specifically waived the need for consent.

### Study population

In the routine diagnostic practice at the University Hospital of North Norway (UNN), the HPV E6/E7 mRNA test PreTect HPV-Proofer (detecting E6/E7 mRNA from the HPV types 16, 18, 31, 33 and 45; (NorChip AS, Klokkarstua, Norway) is used in the triage of women with a Pap-smear showing ASC-US or LSIL and in post-colposcopy follow-up after negative biopsy. The Department of Clinical Pathology receives cervical smears from the population of Troms and Finnmark County. Approximately 23 000 cervical smears are analyzed each year. In 2005–2009, smears from 63 740 women and cervical biopsies from 6 027 women aged 25–69 years were processed. A total of 1 484 women had the cytological diagnoses ASC-H, HSIL or ASC-US/LSIL with a positive HPV mRNA result.

### Exclusion criteria

**Figure 1:**
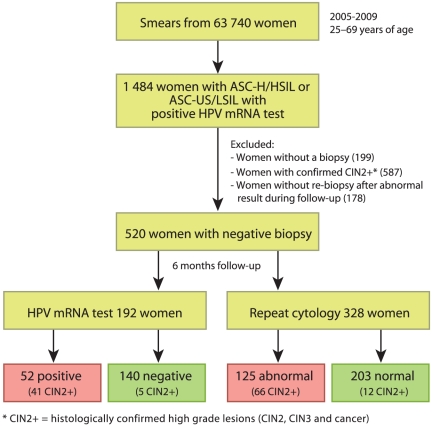
Flow chart showing the two study populations.

The following patients were excluded from the study: women without a biopsy (n = 199); women with CIN2+ on the first biopsy who were referred to treatment (n = 587); and women with one or several abnormal Pap-smears and/or a positive HPV-test at post-colposcopy follow-up but without re-biopsy (n = 178, Figure 1),

Considering the criteria above, 520 women with negative or low-grade biopsy were included in the study. Among these, a liquid based cytology (LBC) follow-up sample was taken for 192 women (36.9%), allowing for additional HPV mRNA testing (HPV group). From the other 328 women (63.1%), a conventional Pap-smear was received, not allowing for HPV analysis (cytology only group, see Figure 1 and Table 1). The result of the follow-up was compared to subsequent histology up to December 2010.

**Table 1:** Total number of women with negative cervical biopsy and later histologically confirmed CIN2+ by cytological result of post-colposcopy cytology.

Follow-up cytology	Number of women	Number of women with CIN2+	PPV1) (%)	95% CI
NIML2)	304	17	5.6	3.0, 8.2
ASC-US	73	19	26.0	16.0, 36.1
LSIL	66	27	40.9	29.0, 52.8
ASC-H	44	33	75.0	62.2, 87.8
HSIL	33	28	84.4	72.6, 97.1
Total	520	124	23.8	20.1, 27.5

1) PPV = Positive predictive value for CIN2+.

2) NIML = Negative for intraepithelial lesion or malignancy.

### Cytology and HPV testing

In LBC, cells were extracted from the ThinPrep® 2000 (Cytyc Corporation, Marlborough, MA, USA) for cytological examination. Of the remnant liquid with cervical cells, DNA/RNA was isolated from 5 ml sample, eluted in 50 µl elution buffer and analyzed with PreTect HPV-Proofer. The mRNA testing was performed according to the manufacturer's instructions (NorChip AS) and in accordance with national guidelines for HPV testing [8].

### Diagnostic database

Cytological and histological diagnoses were obtained from the diagnostic database (SymPathy) at the Department of Clinical Pathology, UNN. Biopsies were evaluated by experienced pathologists and histological results were reported using CIN terminology [22]. In Norway, the threshold for treatment by conization or LLETZ (large loop excision of the transformation zone) is CIN2. Women with benign or CIN1 histology are advised to be followed up with a new Pap-smear and HPV testing after 6–12 months [8]. Biopsies with uncertain cellular changes are analyzed with p16^INK4a^ immunostaining (CINtec® Histology, MTM, Heidelberg, Germany) in order to detect occult CIN lesions.

### Study endpoint

Outcome assessment was based on the histological result of biopsies, where CIN2+ was considered as the target disease and CIN1 and CIN0 (no CIN) were considered as absence of disease. Moreover, women with a complete negative follow-up (two negative post-colposcopy Pap-smears or double negative liquid based cytology (LBC) and HPV mRNA result) were assumed to be free of disease. The clinical sensitivity, specificity, positive predictive value (PPV) and negative predictive value (NPV) were calculated in 2×2 tables for post-colposcopy cytology (with cut-off ASC-US+ and ASC-H+, respectively), for the HPV-test alone and HPV mRNA test in combination with cytology (with cut-off ASC-US+ and ASC-H+).

### Statistical analysis

Pearson's Chi square was used to assess associations between test results and final disease status. To assess differences in accuracy, ratios of the sensitivity, specificity and PPV of mRNA testing versus repeat cytology (and their 95% confidence intervals) were computed. The McNemar Chi square test was used for comparisons in sensitivity and specificity in the second follow-up group where both the RNA test and repeat cytology were applied. The Anova test was used to assess differences in average age of the women in the cytology only group and in the HPV-test group. The statistical computations were performed with the software R version 2.9.0 (2009-04-17), http://www.r-project.org/. P-value<0.05 was considered statistically significant.

## Results

In Table 1, outcomes by post-colposcopy cytology 6 months after the negative biopsy are presented for all the 520 women included. Furthermore, the PPVs for the different cytological follow-up results are presented.

Of the 328 women in the post-colposcopy cytology only group (Tables 2 and 3), 78 women (23.8%) had CIN2+. 207 women were not referred to histology due to two normal post-colposcopy cytology results considered as equivalent to “no disease”, and 43 women had negative biopsy (normal or CIN1). Altogether, 250 women had a negative screening result in follow-up. The accuracy for CIN2+ of triage with post-colposcopy cytology with cut-off ASC-US+ is presented in Table 2. The estimates of sensitivity, specificity, PPV and NPV were 84.6%, 76.4%, 52.8% and 94.1%, respectively. With cut-off ASC-H+, the sensitivity was 53.8%, the specificity 96.4%, the PPV 82.4% and the NPV 87.0% (Table 3).

**Table 2:** Outcome for the 328 women in the cytology only group if triaged with post colposcopy cytology when cut-off is ASC-US+.

Cytological findings	Outcome				%	95% CI
	CIN2+1)	<CIN22)	Total3)	Sensitivity	84.6	76.6, 92.6
ASC-US+	66	59	125	Specificity	76.4	71.1, 81.7
NILM4)	12	191	203	PPV	52.8	44.0, 61.6
Total	78	250	328	NPV	94.1	90.8, 97.4

1) Prevalence CIN2+ 23.8% (95% CI: 19.2–28.4).

2) <CIN2 includes women with a histological diagnosis of CIN1 or CIN0 and women referred back to screening because of two normal post colposcopy smear.

3) Chi square = 91.3, p<0.001.

4) NILM = Negative for intraepithelial lesion or malignancy.

**Table 3:** Outcome for the 328 women in the cytology only group if triaged with post-colposcopy cytology when cut-off is ASC-H+.

Cytological findings	Outcome				%	95% CI
	CIN2+	<CIN2	Total1)	Sensitivity	53.8	42.8, 64.9
ASC-H+	42	9	51	Specificity	96.4	94.1, 98.7
<ASC-H	36	241	277	PPV	82.4	71.9, 92.8
Total	78	250	328	NPV	87.0	83.0, 91.0

1) Chi square = 114.3, p<0.001.

**Figure 2:**
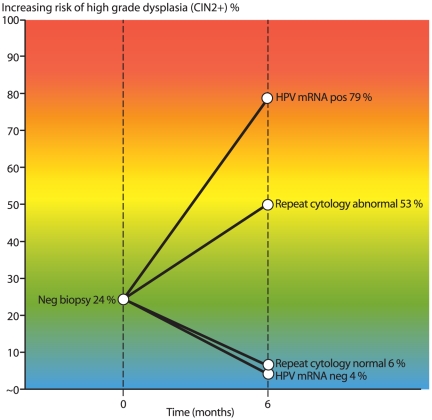
The detection rate of CIN2+ in follow-up of women with negative cervical biopsy by HPV mRNA positive/negative versus cytology abnormal/normal.

Of the 192 women with post-colposcopy HPV mRNA test (Tables 4, 5, and 6), 46 women (24.0%) had CIN2+. 120 women were not referred to histology due to double negative cytology and HPV result considered as equivalent to “no disease”, and 26 women had negative biopsy (normal or CIN1). Altogether, 146 had negative screening result in follow-up. The accuracy parameters for post-colposcopy follow-up with HPV mRNA are shown in Table 4. The estimates of sensitivity, specificity, PPV and NPV were 89.1%, 92.5%, 78.8%, and 96.4%, respectively. The ratios of sensitivity, specificity and PPV of HPV mRNA testing (in the 2^nd^ group) compared to repeat cytology (in the 1^st^ group) for finding CIN2+ was 1.05 (95% CI: 0.92–1.21), 1.21 (95% CI: 1.12–1.32), and 1.49 (95% CI: 1.20–1.86), respectively. These findings were consistent with the intra-group comparison of mRNA testing versus repeat cytology at ASC-US+ cutoff: equal sensitivity (p-value for McNemar's Chi square = 1.00) and superior specificity of mRNA testing (p-value for McNemar's Chi2<0.001). The detection rate of CIN2+ in follow-up of women with negative biopsy by HPV mRNA positive/negative versus cytology abnormal/normal is illustrated in Figure 2.

**Table 4:** Outcome for the 192 women in the HPV group if triaged by post-colposcopy HPV mRNA only.

HPV mRNA result	Outcome				%	95% CI
	CIN2+1)	<CIN22)	Total3)	Sensitivity	89.1	80.1, 98.1
Positive	41	11	52	Specificity	92.5	88.2, 96.7
Negative	5	135	140	PPV	78.8	67.7, 89.9
Total	46	146	192	NPV	96.4	93.3, 99.5

1) Prevalence CIN2+ 24.0% (95% CI: 17.9–30.0).

2) <CIN2 includes women with a histological diagnosis of CIN1 or CIN0 and women referred back to screening because of two normal post-colposcopy smears or normal LBC and a negative HPV result.

3) Chi square = 117.9, p<0.001.

**Table 5:** Outcome for the 192 women in the HPV group if triaged by post-colposcopy HPV mRNA combined with cytology when cut-off is ASC-US+.

Combination result	Outcome				%	95% CI
	CIN2+	<CIN2	Total1)	Sensitivity	97.8	93.6, 100.0
Positive	45	54	99	Specificity	63.0	55.2, 70.4
Negative	1	92	93	PPV	45.5	35.6, 55.3
Total	46	146	192	NPV	98.9	96.8, 100.0

1) Chi square = 50.7, p<0.001.

**Table 6:** Outcome for the 192 women in the HPV group if triaged with post-colposcopy HPV mRNA combined with cytology when cut-off is ASC-H+.

Combination result	Outcome				%	95% CI
	CIN2+	<CIN2	Total1)	Sensitivity	93.5	86.3, 100.0
Positive	43	16	59	Specificity	89.0	84.0, 94.1
Negative	3	130	133	PPV	72.9	61.5, 84.2
Total	46	146	192	NPV	97.7	95.2, 100.0

1) Chi square = 111.9, p<0.001.

HPV mRNA test in combination with cytology with cut-off ASC-US+ had a sensitivity of 97.8%, specificity of 63.0%, PPV of 45.5% and NPV of 98,9% (Table 5). The HPV mRNA test in combination with cytology with cut-off ASC-H+ had sensitivity 93.5%, specificity 89.0%, PPV 72.9% and NPV of 97.7% (Table 6).

Of the 520 women with negative biopsy, 124 women (23.8%) had CIN2+, 72 women (13.8%) had CIN3+ and seven women (1.3%) had cervical cancer in follow-up (Table 7). Five of the seven women with cervical cancer were above 40 years of age (data not shown).

**Table 7:** Diagnosis of the first cervical biopsy compared to the most severe histological diagnosis.

Diagnosis of first biopsy	Most severe histological diagnosis					
	Normal	CIN1	CIN2	CIN3	CxCa	Total
Normal1)	212	6	27	40	4	289
CIN12)	-	178	25	25	3	231
Total	212	184	52	65	7	520

1) Of the women with normal biopsy 24.6% (95% CI: 19.6–29.5) had CIN2+ in follow-up.

2) Of the women with CIN1 biopsy 22.9% (95% CI: 17.5–28.4) had CIN2+ in follow-up.

Of the 192 women with post-colposcopy HPV mRNA test, one woman (0.5%, 95% CI: 0.0–1.5) had cervical cancer. The woman was HPV mRNA positive for HPV type 33. Of the 328 women in the post-colposcopy cytology only group, six women (1.8%, 95% CI: 0.4–3.3) had cervical cancer. Five of the six women had an abnormal cytology. One woman had normal cytology (negative for intraepithelial lesion or malignancy - NILM), but her cervical cancer was detected by hysterectomy with indication uterine leiomyoma (data not shown).

## Discussion

Colposcopy and colposcopically-guided biopsies do not have optimal sensitivity for detection of CIN2+. Of the 520 women with negative biopsy, seven women actually had cervical cancer. For these women, the first biopsy or histological slide may not have been representative for the underlying disease. Alternatively, a microlesion difficult to detect by colposcopy, colposcopy-directed biopsies or by evaluation of histology may have been present. Nevertheless, this emphasizes the need for additional methods in order to increase the overall sensitivity of the screening algorithm in the prevention of cervical cancer.

The current poor performance of colposcopic impression, colposcopically-guided biopsies and histological diagnosis limits the potential benefit of a very sensitive screening test [23]. In Norway, for women with high grade cytology or HPV positive low grade cytology, it is recommended two colposcopically-guided biopsies and two random biopsies. If the colposcopy is normal, biopsies from all four quadrants of the cervix is recommended, in addition to an endocervical curette. Still, a considerable number of women with a negative first biopsy have CIN2+ in the follow-up. In our material 23.8% of the women with negative first biopsy actually had CIN2+. Women with negative colposcopy and/or negative biopsies require follow-up before resumption of routine screening.

The main cause of invasive cervical cancer is the deregulated and persistent production of HPV E6 and E7 oncoproteins [24]. Hence, HPV E6/E7 mRNA is a rational target for detecting HPV infections leading to cellular transformation. PreTect HPV-Proofer detects E6/E7 mRNA of the five main high-risk HPV types 16, 18, 31, 33, and 45, which cause 86% of cervical cancers in Europe [1], [25]. Due to the higher clinical specificity and PPV of this method compared to other HPV tests [13]–[18], [26], this was the method of choice at our hospital when HPV testing was introduced in triage of women with minor cytological lesions and in follow-up of women with negative biopsy.

When summarizing results from all women included in the study (Table 1), 124 of 520 women (23.8%) had CIN2+ confirmed by biopsy in follow-up. The PPV of post-colposcopy ASC-US or LSIL in follow-up after negative biopsy were relatively low. Previously, before HPV testing was included in clinical practice, women with post-colposcopy ASC-US/LSIL were referred to follow-up by a new cytology after 6–12 months. In current practice, this is still the case for women with repeated ASC-US/LSIL and a negative HPV mRNA result. Women with a positive post-colposcopy HPV result however, are referred directly to a new colposcopy with biopsy [8]. A high PPV of the HPV test is therefore important in order to avoid unnecessary follow-up, both with regard to follow-up costs to the health care system and with regard to unnecessary psychological stress for the patients. In addition, it is known that HPV tests identify lesions not found by cytology and thereby contribute in increasing the overall clinical sensitivity of the screening program [27]–[33].

It is also important to have in mind that in clinical practice, the cytological diagnosis is commonly influenced by the HPV result. We see a tendency that a preliminary diagnosis of for example normal or ASC-US is upgraded when a positive HPV mRNA result is acknowledged. As a result, the clinical sensitivity of cytology as such will be over-estimated. In order to get two groups for comparison, a study with two separate arms was conducted. One arm with post-colposcopy repeat cytology only and one arm with both repeat cytology and HPV test. National guidelines recommend that a woman with negative biopsy should be followed up with repeat cytology and an HPV test after 6–12 months. The HPV-test however is not always performed due to use of conventional Pap-smears instead of LBC, a method not allowing for HPV testing. This made it possible to get a separate arm with repeat cytology only. The HPV test result is objective and not dependent on the cytological diagnosis. Women referred to histology based on the cytological result alone (i.e., having a negative HPV result) made it possible to calculate clinical properties also including HPV negative results.

Until 2006, only conventional Pap-smears were used at our hospital. When HPV-testing was introduced, liquid based cytology was recommended, but still almost half of the samples for repeat cytology are conventional Pap-smears. A limitation of the study is the absence of formal randomization, which can result in imbalanced groups. Still, one argument about comparability is the similar age and similar prevalences of CIN2+. In the group with cytology only, follow-up and in the HPV group, the average age was 41.1 and 42.4 years, respectively (p = 0.235). The median age was 38 and 39 years, respectively. The prevalence of CIN2+ was similar in the two groups (23.8% and 24.0%, respectively).

Referral to biopsy in the HPV-category is based on both the cytology and the HPV result and therefore does not represent the true number of women referred to biopsy based on HPV testing alone. For example, women with HSIL and ASC-H were always referred to biopsy independent of the HPV result. Women with ASC-US and LSIL were followed with new cytology after 12 months if the HPV mRNA test was negative.

With regard to follow-up cytology, statistical parameters were calculated both at cut-off ASC-US+ and ASC-H+. The reason for this is different follow-up in the two groups. Women with ASC-H+ are referred to colposcopy and biopsy. Women with ASC-US and LSIL are followed up with cytology and HPV-testing. Post-colposcopy cytology with cut-off ASC-US+ has a high sensitivity (84.6%) for CIN2+, but only a moderate specificity (76.4%). In contrast, repeat cytology with cut-off ASC-H+ has a low sensitivity (53.8%), but a high specificity (96.4%). The combination of HPV mRNA testing and cytology with cut-off ASC-H+ has both high sensitivity (93.5%) and high specificity (89.0%).

The HPV mRNA data for women above and under 40 years of age were analyzed separately. This was done both because older age is associated with lower rates of regression of lesions, a higher degree of recurrence after treatment of precancer and the risk of obstetrical adverse effects is of less importance due to the lower rate of planned children [19], [20], [34]–[37].

For the 97 women under 40 years of age with HPV mRNA test, the estimates of sensitivity, specificity, PPV and NPV were 88.9%, 91.4%, 80.0% and 95.5%, respectively. For the 95 women above 40 years of age with HPV mRNA test, the estimates of sensitivity, specificity, PPV and NPV were 89.5%, 93.4%, 77.3% and 97.3%, respectively (Table 8).

**Table 8:** Outcome for the 97 women under 40 years and the 95 women above 40 years in the HPV group if triaged with post-colposcopy HPV mRNA only.

HPV mRNA result	Age<40 years			Age>40 years		
	CIN2+	<CIN2	Total1)	CIN2+	<CIN2	Total2)
Positive	24	6	30	17	5	22
Negative	3	64	67	2	71	73
Total	27	70	97	19	76	95

	%	95% CI	%	95% CI
Sensitivity	88.9	77.0, 100.0	89.5	75.7, 100.0
Specificity	91.4	84.9, 98.0	93.4	87.8, 99.0
PPV	80.0	65.7, 94.3	77.3	59.8, 94.8
NPV	95.5	90.6, 100.0	97.3	93.5, 100.0
Prevalence CIN2+	27.8	18.9, 36.8	20.0	12.0, 28.0

1) Chi square = 58.8, p<0.001.

2) Chi square = 58.7, p<0.001.

In the present study, the HPV mRNA test shows a specificity of 92.5%. Previous data on HPV mRNA testing in the triage of ASC-US and LSIL [8], [12], [21] show that the HPV mRNA test, due to a high specificity and positive predictive value (PPV), is valuable in the triage of women with minor cytological cervical lesions as it gives important information in terms of further follow-up. In fact, when the HPV result is positive, the data suggest direct treatment for women above 40 years of age or for women with a concurrent cytological HSIL diagnosis, contributing to better clinical safety for these patients [8]. Benevolo et al reported 80% PPV for CIN2+ in women with positive HPV mRNA and HSIL cytology [38]. Schiffman et al recommend direct treatment if the risk of CIN3+ is high, for example 80–90% [23]. In the present study, the PPV for CIN2+ for HPV mRNA positive in follow-up of women with negative biopsy was 78.8%; the PPV was 80.0% for women under 40 years of age, and 77.3% for women above 40 years of age. Zuchna et al reported that colposcopy and biopsy had 66.2% sensitivity of CIN2+ [10]. When a follow-up test for women with negative biopsy has a high PPV for CIN2+, direct treatment is more suitable than re-biopsies for women older than 40 years of age if the triage test is positive. Given the impact of cervical treatment (such as LLETZ or conization) on pregnancy outcomes [19], [20], [35], [37], [39], [40], cervical treatment is more critical for women planning to have children. In addition, if a high-grade lesion is found in a woman above 40 years of age, there is a higher chance that the lesion has been present for a longer period of time, and at higher risk of progression [34], [41].

Women with negative biopsy require follow-up before resumption of routine screening. In the present study, the HPV mRNA test is as sensitive and significantly more specific than repeat cytology at cut-off ASC-US+ in follow-up of women with negative biopsy. Thus, the study findings provide evidence that supports our hypothesis that HPV E6/E7 mRNA testing is more specific than follow-up cytology without loss in sensitivity in postdiagnostic management of women who have a negative biopsy. In addition, the HPV mRNA test has a significantly higher PPV than cytology alone. These data indicate that the HPV mRNA test is a better follow-up test than cytology for women with negative biopsy. When the follow-up test is HPV mRNA positive, our data suggest direct treatment for women above 40 years of age contributing to a better clinical safety for these patients. The use of HPV mRNA test can reduce the time from a false negative biopsy to diagnosis and treatment for CIN2+. Further studies are needed in order to reveal whether post-colposcopy HPV mRNA testing and E6/E7 detection may replace cytology in follow-up of women with negative biopsy.
